# PM Origin or Exposure Duration? Health Hazards from PM-Bound Mercury and PM-Bound PAHs among Students and Lecturers

**DOI:** 10.3390/ijerph15020316

**Published:** 2018-02-12

**Authors:** Grzegorz Majewski, Kamila Widziewicz, Wioletta Rogula-Kozłowska, Patrycja Rogula-Kopiec, Karolina Kociszewska, Tomasz Rozbicki, Małgorzata Majder-Łopatka, Mariusz Niemczyk

**Affiliations:** 1 Faculty of Civil and Environmental Engineering, Warsaw University of Life Sciences, 166 Nowoursynowska St., 02-776 Warsaw, Poland; grzegorz_majewski@sggw.pl (G.M.); karolina_kociszewska@sggw.pl (K.K.); Tomasz_rozbicki@sggw.pl (T.R.); 2 Institute of Environmental Engineering, Polish Academy of Sciences, 34 M. Skłodowska-Curie St., 41-819 Zabrze, Poland; wioletta.rogula-kozlowska@ipis.zabrze.pl (W.R.-K.); patrycja.rogula-kopiec@ipis.zabrze.pl (P.R.-K.); 3 Faculty of Fire Safety Engineering, The Main School of Fire Service, 52/54 Słowackiego St., 01-629 Warsaw, Poland; mmajder@sgsp.edu.pl; 4 Department of Immunology, Transplant Medicine and Internal Diseases, Medical University of Warsaw, Nowogrodzka 59, 02-006 Warsaw, Poland; mariuszniemczyk@wp.pl

**Keywords:** indoor air quality, mercury, PAHs, health risk assessment, universities, lecture rooms, Poland

## Abstract

This study assessed inhalation exposure to particulate matter (PM_1_)-bound mercury (Hg_p_) and PM_1_-bound polycyclic aromatic hydrocarbons (PAHs) among university students. For this purpose, simultaneous indoor (I) and outdoor (O) measurements were taken from two Polish technical universities (in Gliwice and Warsaw) located in distinct areas with respect to ambient concentrations and major sources of PM. The indoor geometric mean concentrations of Hg_p_ were found to be 1.46 pg·m^−3^ and 6.38 pg·m^−3^ in Warsaw and Gliwice, while the corresponding outdoor concentrations were slightly lower at 1.38 pg·m^−3^ and 3.03 pg·m^−3^, respectively. A distinct pattern was found with respect to PAH concentrations with estimated I/O values of 22.2 ng·m^−3^/22.5 ng·m^−3^ in Gliwice and 10.9 ng·m^−3^/11.12 ng·m^−3^ in Warsaw. Hazard quotients (HQs) as a result of exposure to Hg_p_ for students aged 21 ranged from 3.47 × 10^−5^ (Warsaw) to 1.3 × 10^−4^ (Gliwice) in terms of reasonable maximum exposure (RME). The non-cancer human health risk value related to Hg_p_ exposure was thus found to be below the acceptable risk level value of 1.0 given by the US EPA. Daily exposure values for lecture hall occupants, adjusted to the benzo(a)pyrene (BaP) toxicity equivalent (BaP_eq_), were 2.9 and 1.02 ng·m^−3^ for the Gliwice and Warsaw students, respectively. The incremental lifetime cancer risk (ILCR) values with respect to exposure to PM_1_-bound PAHs during the students’ time of study were 5.49 × 10^−8^ (Warsaw) and 1.43 × 10^−7^ (Gliwice). Thus, students’ exposure to indoor PAHs does not lead to increased risk of lung cancer.

## 1. Introduction

In many parts of the world, humans are constantly being exposed to the excessive concentrations of particulate matter (PM)-bound toxic compounds such as polycyclic aromatic hydrocarbons (PAHs) or particulate mercury (Hg_p_). The European Parliament faces severe and growing challenges in its efforts to meet air quality standards with respect to PAHs and mercury pollution [1,2]. Increased amounts of mercury and PAHs in atmospheric air have been widely attributed to a global increase in fossil fuel emissions rather than industrial or traffic sources. This is in accordance with the results from different source apportionment studies [3,4,5] signaling coal and solid fuel combustion as the main sources of mercury and PAHs released into the atmosphere. This is, however, not the case for gaseous elemental mercury (GEM) for which concentrations at the receptor sites are caused by oxidation reactions, boundary layer mixing, and flux from surfaces [3] rather than anthropogenic sources. Both atmospheric PAHs and mercury are partitioned between the particulate and the gaseous phases. Due to a low vapor pressure and low water solubility, the most hazardous carcinogenic 5-; 6- and 7-ring PAH species tend to adhere to particles rather than exist in the gaseous phase [6,7]. In contrast to particle-bound PAHs [8], Hg_p_ represents a small part of the total mass of ambient mercury [9,10]. However, the presence of even small amounts of aerosol-bound Hg_p_ and PM-bound PAH species in the air is very hazardous since those compounds have the potential to be highly toxic [11,12]. The negative health effects resulting from the exposure to these compounds are of particular concern in environments where people spend the majority of their time—inside buildings. Good examples of such indoor environments are university campuses with dense populations of students.

Indoor air pollution in lecture halls and dorm rooms can cause different disorders among university students who spend more of their time at university than in any other indoor environment except their homes. Such pollution conditions can be due to malfunctioning ventilation systems that cause poor air quality inside lecture halls [13], high air temperature [14], humidity [15], or the presence of indoor pollution sources [16,17]. Exposure to those conditions can lead to health risks such as upper airway irritations (which can distract students), carbon monoxide poisoning [18], blood pressure problems [19], and even asthma [20]. Due to varying levels of sensitivity among individuals, indoor air quality (IAQ) problems may affect only a selected group of university occupants. On comparing numerous recent studies on indoor air pollution within universities [21,22,23,24] and student campuses (lecture halls, theatres, canteens), we found only one foreign example of research raising the subject of student exposure to mercury or PAH compounds [25]. There are no epidemiological studies concerning student exposure to indoor PAHs and Hg_p_ within university buildings. The existing research relates mainly to concentration measurements than to health effects. For example, Peng, et al. reported values ranging from 9.84 μg·g^−1^ to 21.44 μg·g^−1^ for PAHs in dust samples collected from lecture theatres in Shanghai, and values ranging from 9.63 μg·g^−1^ to 44.13 μg·g^−1^ for samples from dining halls [25]. An indoor/outdoor ratio of BaP estimated at 0.82 (indicating its outdoor origin) was found in a primary school in Taranto (Italy) [26]. Higher PAH concentrations were observed when schools were downwind of a steel plant. Another study indicated that some pollution sources such as major roads, dust resuspension from ground surfaces around schools, and biomass use in the areas around schools may be important determinants of exposure [27]. In other words, depending on the proximity to major emission sources (such as industrial facilities, roads, landfills, and so on), PM-bound PAHs can penetrate buildings and result in elevated indoor concentrations [28]. Both indoor and outdoor sources of Hg_p_ and PAHs could be important with respect to student exposure, causing different harmful effects. The division of inhalation risks between indoor and outdoor exposures is strictly connected not only with differences in pollution levels between those environments but also with specific social activities and types of ventilation in the university buildings.

Recently, we estimated PM_1_-bound Hg and PAH concentrations from regular measurements during our university campaign [29,30]. The mentioned studies poorly represent the personal exposure of students since they focus only on the differences in the distribution of those compounds between the indoor and outdoor environments. Since the level of exposure depends on the fractions of time that individuals spend in various indoor and outdoor environments, as well as the concentrations of the outdoor air pollutants (which by infiltration migrate into buildings), personal exposure to ambient air pollutants occurring in both indoor and outdoor environments should be taken into account [31,32]. Therefore, the aim of our study was to calculate and assess the differences in the exposure of students to PM-bound mercury and PAH pollution during their time at university and in function of their personal activities. For this purpose, we calculated the students’ exposure by integrating the measurements of indoor and outdoor concentrations of PM-bound Hg and PAHs in lecture halls with the statistical data concerning their average use of time [33]. The second question put in this study was how the elongation of time spent inside university buildings can modify exposure. The answer was delivered by considering the occupational exposure of university lecturers. Understanding the activity of students and lecturers and their times spent inside and outside the university is a first step in identifying the actions necessary to reduce or avoid the adverse impacts of air pollution on health.

## 2. Methods

### 2.1. Location Description

Particulate matter samples with a particle diameters smaller than 1 micron—(PM_1_) were collected from a third-floor roof-top of the Silesian University of Technology New Technologies Center (CNT) located in Gliwice and from lecture halls at the Warsaw University of Life Sciences (WULS-SGGW). In the course of this study, we took into consideration the geographical location of the university buildings and all correlated parameters regarding the environment close to their location (e.g., building typologies, distance from the main outdoor pollution sources such as traffic sources, the presence of green-zone areas, urbanization levels, etc.).

The CNT is located at the northeast N–E (50°17′36.5″ N–18°40′57.1″ E)-oriented Konarskiego 18 St., and is surrounded by old Silesian University of Technology buildings, and lies just next to the DTŚ highway. The WULS-SGGW buildings (52°09′40.1″ N–21°02′46.9″ E) are placed in a relatively densely-built area within the city. The distance from university buildings to the nearest residential area, where solid fuels might be combusted for heating purposes, is 850 m in the case of Warsaw and 350 m for Gliwice. The nearest coal-fired power plants are located about 4 km from the SGGW buildings and 2.5 km from the CNT buildings. The CNT building is covered by glass, without any operable windows. Therefore, there is no access to fresh air and the ventilation is supplied artificially by the air conditioning system. In contrast, the SGGW buildings rely on natural ventilation, which is left to the room users. During the sampling process, the windows were closed each time. Both of the monitored rooms were equipped with standard furniture including lecture hall tables made of wood with seating fixed to the floor, a blackboard, and an overhead projector. A detailed description of the sampling location along with the situational plan of the lecture halls can be found in [29,30].

### 2.2. PM_1_ Sample Collection

Particulate matter samples (PM_1_ fraction) were collected at the Warsaw and Gliwice sampling sites during the warm season, between 20 April 2015 and 1 June 2015. In total, 120 daily (24-h) indoor and outdoor samples were simultaneously taken using 47-mm Q-MA quartz filters (Whatman, Maidstone, UK). In some regions of Poland, especially in urban areas, the yearly pattern of PM pollution is blurred by winter maxima (so-called smog episodes). This is especially true during cold weather where normal PM concentrations are obscured by municipal emissions from residential heating, primarily from hard coal combustion [34,35]. Therefore, in this study, we shortened the sampling campaign to the summer period when the trend of PM concentration is more stable. Single campaigns lasted from Monday to Friday during lecture hours. Both sampling locations were equipped with the same standard PM_1_ sampling sets. They were comprised of four MVS (Medium Volume Sampler) (2.3 m^3^·h^−1^) samplers: two Atmoservice’s in Warsaw and two Leckel’s KFG in Gliwice, equipped with an exchangeable jet (TSI company) removing particles with diameters greater than 1 µm from the air stream. Before exposure, pre-weighted clean quartz filters directed for PAH analysis were heated at 650 °C for 2 h, then conditioned in a weighing room (48 h; relative air humidity 45% ± 5%; air temperature 20 ± 2 °C). After exposure, the filters were weighed twice (at 24 h intervals) on a Mettler Toledo AT microbalance (with a resolution of 2 µg) equipped with a Haug U-ionizer. The same procedure was used for the filters for Hg analysis (excluding the heating step). To prevent Hg re-volatilization, the filters were put into containers after weighing, tightly enveloped in aluminum foil, and kept in a refrigerator (2–4 °C) until the analysis. The procedures for conditioning, weighing, storage, and transport of all samples, including blank samples, were done in agreement with the QA/QC (Quality Assurance/Quality Control) procedures of the reference method for gravimetric measurements [36]. The weighting accuracy, determined as three standard deviations from the mean and obtained from ten weightings of a blank filter (conditioning performed every 48 h), was 20.5 µg. The method of preparing filters for chemical tests and analyses, as well as the methodology of gravimetric analysis, was previously described by [29,30].

### 2.3. Hg Analysis

PM_1_-bound mercury content was determined using an MA-2 analyzer (Nippon Instr. Co., Tokyo, Japan). In total, 60 Hg_p_ samples were determined (30 samples from the indoor and 30 samples from the outdoor environments). Determination of the Hg_p_ content was based on the thermal decomposition of the PM_1_ sample and the detection of the released Hg vapor by cold vapor atomic absorption spectrometry (CVAAS). The PM_1_ sample (a 1.5-cm^2^ section of an exposed filter) and additives were placed in a quartz boat and heated to 700 °C in mercury-free air in the decomposition furnace. All analyses of PM_1_-bound mercury, quality control, and method validity were performed according to the description presented by [29].

### 2.4. PAH Analysis

The concentrations of the following PM_1_-bound PAHs were determined chromatographically (GC-MS) after dichloromethane (CH_2_Cl_2_) extraction in an ultrasonic bath: naphthalene (Na), acenaphthylene (Acy), acenaphthene (Ace), fluorene (Flu), phenantrene (Ph), anthracene (An), fluoranthene (Fl), pyrene (Py), benzo[a]anthracene (BaA), chrysene (Ch), benzo[b]fluoranthene (BbF), benzo[k]fluoranthene (BkF), benzo[a]pyrene (BaP), indeno[1,2,3-cd]pyrene (IP), dibenzo[ah]anthracene (DBA), and benzo[ghi]perylene (BghiP). In total, 60 PAH samples were determined (30 samples from the indoor and 30 samples from the outdoor environments). Information concerning PM-bound PAH extraction, their analysis, detection limits, and method validation can be found in [30].

Both the recovery rates and the standard deviations of the studied PAHs and Hg in the analyzed samples can be found in the previously mentioned papers of the author [29,30].

### 2.5. Exposure Assessment and Risk Analysis

An important element in the methodological framework used in this work for calculating exposure to PM_1_-bound PAHs and Hg_p_ was the study of the students’ average use of time. This “use of time framework,” which is the basis for the provided studies, was approximated based on the results presented and released by the U.S. Bureau of Labor Statistics (American Time Use Survey; [33])*.* Since the Polish education system is quite similar to that of the US and the amount of time to finish a Polish Eng. or Master of Science degree is equal to the U.S. Master or Bachelor of Engineering degree, such an approximation seems reasonable. The cited report includes data from the U.S. population (age 15 to 49) attending college or university in the US and provides information about how students spend their time. In this work, the relationships between the presence of students in university lecture halls and the level of exposure to PM_1_-bound compounds are presented by the time of day. The activities presented in the survey consist of eight major activities. These activities (with the proportion of the day spent for each given as a percentage in brackets) are as follows: sleeping (~37%), leisure and sports (~17%), educational activities (~15%); working and related activities (~10%); traveling (~6%); eating and drinking (~4%); grooming (~3%); and other (~9%). Among the eight activities, the largest share is allocated to sleep (36%); while college/work-related activities (and therefore the sum of the educational and work-related activities) represent ~25%. Besides work-related activities, social-recreational activities—for example, involving leisure and sport—represent a relatively high proportion of time. For students living at the campus (for example, in dormitories), sporting activities usually take place in the interiors of closed sports facilities such as wrestling rooms, etc. It should also be noted that the average duration of work activities is more than twice the duration of recreation plus sports activities. To determine the daily changes in the exposure to PM_1_-bound PAHs and Hg_p_, the students’ days were divided into eight time intervals (activity profile) j = 1, … 8, where each j_i_ from the j dataset was assigned to a specific indoor (I) or outdoor (O) environment (Table 1). This clearly shows how students are engaged in given activities in each hourly time frame of a day, and how these activities are aggregated between indoor and outdoor environments.

When calculating the share of time the hypothetical students spend in the indoor environment, we obtained a figure of ~85% of the total daily time, which is in good agreement with the data from [37]. Based on a simple activity schedule, together with indoor/outdoor concentration data for individual university buildings, the active dose of inhaled PM_1_-bound mercury (Equation (1)) and PM_1_-bound PAH compounds (Equation (5)) was calculated and the exposure profiles were further developed. To determine the daily changes in student exposures, it was assumed that the daily dose of inhaled mercury and PAHs was equal to their airborne concentrations in specific indoor or outdoor environments.

#### 2.5.1. The Non-Carcinogenic Health Risk from Mercury Exposure

Following the United States Environmental Protection Agency [38], the estimation of the exposure concentrations (ECs) for non-cancer effects was initiated through assessment of scenario-specific exposure patterns. Due to the fact that students spend approximately 85% of their time indoors (where Hg concentrations could be even higher than those characteristic of outdoor conditions), we assumed that during their time of study, students were constantly and acutely exposed to the measured Hg_p_ levels. To properly represent the Hg_p_ toxicity value, we set the exposure times (ETs) to 24 h over the period of study (acute toxicity). The uncertainties associated with such a choice are obvious and undisputed. However, in this work, we tried to estimate the most probable scenario, not the worst-case scenario as shown in the example in [39]. In this work, the exposure concentration (EC) is equal to the mercury concentration (CA) in the air. However, to divide this exposure between indoor and outdoor concentrations, we treated the total exposure concentration as the sum of exposures from the outdoor and indoor Hg_p_ concentrations:

(1) $$\mathrm{EC}=\frac{\left(\left({\mathrm{CA}}_{I}\cdot 0.85\mathrm{ET}\right)+\left({\mathrm{CA}}_{O}\cdot 0.15\mathrm{ET}\right)\right)\cdot \mathrm{EF}\cdot \mathrm{ED}}{\mathrm{AT}}$$

where EC (μg·m^−3^) = exposure concentration; CA (μg·m^−3^) = mercury concentration in air; CA_I_ indicates the indoor levels of Hg_p_, and CA_O_ indicates the outdoor concentrations of Hg_p_; ET (hours·day^−1^) = exposure time (24 h·day^−1^); 0.85ET indicates the time students spend in the indoor environment; 0.15ET indicates the time students spend in the outdoor environment; EF (days·year^−1^) = exposure frequency (266 days·year^−1^); ED (years) = exposure duration (5 years); AT (ED in years × 365 (days·year^−1^) × 24 (hours·day^−1^)) = averaging time.

When assessing the risk, we did not include any age-dependent exposure variability. On inclusion in the risk model, the subsequent exposure variables often make the predictions hypothetical. The hazard quotient (HQ), reflecting the overall potential non-carcinogenic effect, was calculated by Equation (2):

(2) $$\mathrm{HQ}=\frac{\mathrm{EC}}{R_{f}C_{i}}\times \left(1000\frac{\text{µ}g}{\mathrm{mg}}\right)$$

where HQ is the hazard quotient = non-carcinogenic risk; R_f_C_i_ is the inhalation reference concentration (mg·m^−3^).

The inhalation reference concentration (R_f_C_i_) for mercury is analogous to the oral reference dose R_f_D and is likewise based on the assumption that thresholds exist for certain toxic effects. In this study, we used an R_f_C_i_ value for elemental mercury of 3 × 10^−4^ mg·m^−3^, as presented in the US EPA and Integrated Risk Information System (IRIS) summary, which can be seen at [40].

According to the US EPA recommendations, HQ values less than unity (<1) indicate no significant health risk/no adverse health effects. Values above one indicate the possibility of non-carcinogenic effects (Risk Assessment Guidance for Superfund [41]). It is, however, worth noting that a revised reference concentration (R_f_C) for mercury vapor in adults already exists [42].

#### 2.5.2. The Carcinogenic Health Risk from PAHs Exposure

The inhalation exposure to the PAH mixture was adjusted by the toxicity equivalent factor (TEF) to the benzo(a)pyrene (BaP) toxicity equivalent (BaP_eq_) independently for indoor (I) and outdoor (O) environments (according to Equations (1) and (2)):

(3) $${\mathrm{BaP}}_{\mathrm{eqI}}=\sum_{i=1}^{n}C_{I}\cdot {\mathrm{TEF}}_{i}$$

(4) $${\mathrm{BaP}}_{\mathrm{eqO}}=\sum_{i=1}^{n}C_{O}\cdot {\mathrm{TEF}}_{i}$$

where the BaP_eqI_ and BaP_eqO_ (carcinogenic equivalent, ng·m^−3^) are the sums of the converted PAH concentrations multiplied by toxic equivalents of BaP presented in Table 2 [43]. The BaP_eq_ for the sum of 16 nonvolatile PAHs is therefore:

(BaP_eq_)∑16PAH = [NAP] × 0.001 + [AcPy] × 0.001 + [Acp] × 0.001 + [Flu] × 0.001 + [PA] × 0.001 + [Ant] × 0.01 + [FL] × 0.001 + [Pyr] × 0.001 + [BaA] × 0.1 + [CHR] × 0.01 + [BbF] × 0.1 + [BkF] × 0.1 + [BaP] × 1 + [IND] × 0.1 + [DBA] × 1 + [BghiP] × 0.01

The health risk for students with respect to inhalation exposure to PAHs was calculated following US EPA recommendations [41] using the deterministic approach. This approach uses point values to produce a point estimate of exposure (in this case the reasonable maximum exposure). The exposure scenario was based on a few assumptions concerning exposure time and frequency, which are listed here.

In Poland, the academic year starts in October and lasts until the end of June, covering on average 266 days of the calendar year (excluding weekends, school holidays, and national holidays). We, therefore, assumed that students are able to spend a maximum of 266 days per year inside the university. The typical exposure duration for students in Poland is equal to the duration of the course of study, which usually lasts 5 years. The first level of study, the so-called engineer’s degree, lasts 3 or 3.5 years depending on the area chosen by the student. This first stage can then be followed by a second step, a Master’s degree, which requires 1.5 or 2 more years of study. The duration of exposure to PM-bound PAHs and Hg_p_ over the lifetime of a student is therefore 5 years, and t is the daily time spent in each environment—in this case, 20.4 h indicates time spent in the indoor environment, while 3.6 h indicates the time that students spend in the outdoor environment. Considering that students usually start studying at the age of 19 (after passing the Matura exam), this group of the population is mostly made up of adults (with ages ranging from 19 to 24 years of age), weighing approximately 70 kg (body weight, BW). To model the inhalation rate (IR) under conditions of light activity, we chose a value of 22.8 m^3^·day^−1^, well characterizing the activity of a sedentary adult, (i.e., while studying) [46].

The daily inhalation exposure level (IEL, ng·day^−1^) to PAHs for students was calculated by multiplying the BaP_eqI_ and BaP_eqO_ concentrations (ng·m^−3^), inhalation rate (IR, m^3^·day^−1^), and daily exposure time span (t, h·day^−1^) as follows (Equation (5)):

(5) $$\mathrm{IEL}=({\mathrm{BaPeq}}_{I}\cdot 0.85t+{\mathrm{BaPeq}}_{O}\cdot 0.15t)\cdot \mathrm{IR}$$

The incremental lifetime cancer risk (ILCR) posed by exposure to PM-bound PAHs was computed following Equation (6). The calculation of the health risk involved the multiplication of IEL by the carcinogenic slope factor (SF) for BaP_eq_ by following Equation (2). Each of the variables in the risk equation was modeled as a point estimate since the usage of specific probability distribution (PDF) functions would be too hypothetical. Equation (6) is given as follows:

(6) $$\mathrm{ILCR}=\frac{{\mathrm{SF}}_{\mathrm{inh}}\cdot \mathrm{IEL}\cdot \mathrm{EF}\cdot \mathrm{ED}}{\mathrm{BW}\cdot \mathrm{AT}\cdot \mathrm{cf}}$$

where ILCR is the incremental lifetime cancer risk of BaP_eq_ exposure (dimensionless); and SF is the inhalation cancer slope factor of BaP (kg·day·mg^−1^), which is used to evaluate the relationship between the concentration of a certain PAH compound and the corresponding cancer risk. In this study, the SF with a geometric mean of 3.14 kg·day·mg^−1^ was used as derived by [47]. The proper description of how the SF value was derived was presented in [48]. IEL represents the BaP_eq_ daily dose (µg·day^−1^); EF is the exposure frequency (day·year^−1^); ED is the exposure duration for adults, here estimated as 5 years; BW is the body weight, here given as 70 (kg) according to [46]; and AT represents the average lifespan with respect to carcinogens averaged for both male and female residents of Poland (males: 73.6 years = 26,864 days; women: 81.6 years = 29,784 days) (Central Statistical Office in Poland [49]). Finally, cf is the conversion factor (10^6^) (ng·mg^−1^). In accordance with the US EPA, a one in a million chance of developing an additional human cancer over a lifetime (ILCR = 10^−6^) was considered to be an acceptable level of risk, whereas a lifetime risk of one in a thousand or greater (ILCR = 10^−3^) was considered to be a serious health threat. Knowing that exposure time is one of the parameters most strongly influencing inhalation risk, we additionally performed exposure calculations for teaching staff with 40 years of work experience. In that scenario of exposure, the duration time (ED) was stretched to 40 years.

## 3. Results

### 3.1. Chemical Characteristics of PM_1_-Bound Compounds in Gliwice and Warsaw

In the present study, sampling and detection of indoor and outdoor PM_1_-bound PAHs and Hg_p_ was performed at two technical universities in Poland. The concentration levels of individual PAH compounds, including low molecular weight (LMW, 2–3 rings) PAHs, high molecular weight (HMW, 4–7 rings) PAHs, and also the sum of ∑16PAH concentrations in indoor and outdoor PM_1_, are listed in Table 3. The average concentration of ∑16PAH congeners in the indoor environment was 12.97 ng·m^−3^ in Gliwice and 6.78 ng·m^−3^ in Warsaw, while the average outdoor concentrations were 14.85 ng·m^−3^ in Gliwice and almost half that value at 8.08 ng·m^−3^ in Warsaw. Those concentration levels were low compared to the PM-bound PAH averages typical for winter periods found in our previous work [35,50]. PM_1_ samples collected in Gliwice contained predominantly benzo[a]pyrene (BaP); benzo[a]anthracene (BaA); fluoranthene (FL); chrysene (CHR); benzo[b]fluoranthene (BbF); and fluorene (Flu); while the samples taken at the Warsaw site were found to be predominantly comprised of fluoranthene (FL); benzo[a]pyrene (BaP); and chrysene (CHR). Indoor and outdoor PM_1_ samples showed slightly different PAH profiles. One example is the fact that concentrations of BbF were dominant in the outdoor air at the Gliwice site (1.90 ng·m^−3^), while the indoor concentrations were not very high, equaling 1.17 ng·m^−3^. More 4- and 5-ring PAHs were detected in the outdoor air as compared to the closed lecture halls, with higher contributions of 4-ring PAHs were found at the university in Gliwice.

The aromatic rings of PAHs influence their toxicity and carcinogenicity. The differences in the number of rings between the indoor and outdoor PM_1_ samples were evaluated. We found that high molecular weight (HMW) PM_1_-bound PAHs (4 + 5 + 6-ring PAHs) accounted for about 78–79% of total PAH concentrations in fine PM samples from lecture halls and 87% of total PAH concentrations in the ambient air (average shares in the Gliwice and Warsaw sites). Among the 16 detected PAHs included in this study, the best known, due to its mutagenic and carcinogenic effects, is benzo(a)pyrene (BaP). For many years BaP was found to be a good and sufficient indicator of human exposure to carcinogenic ambient PAHs. However, since it has been proven that other PAH conglomerates (e.g., dibenzo(a,h)anthracene) may have higher carcinogenic potential, it has become clear that BaP is not a sufficient substitute marker for the carcinogenicity of PAHs. In this study, ∑_PAHscarc_ (the summed up concentrations of carcinogenic PAHs) accounted for approximately 74% of the ∑_PAHs_ indoors and 83% outdoors (values averaged for both sites). BaP represented the greatest proportion of carcinogenic PAHs at the university in Gliwice, accounting for approximately 18% of ∑_PAHscarc_. At the university in Warsaw, fluoranthene made the greatest contribution to the total considered PAHs, on average accounting for 34% of ∑_PAHscarc_.

Due to the origin-related dissimilarities, it was supposed that the I/O ratios of PM-bound Hg should be quite different to those of PAHs. In Gliwice, the concentrations of Hg_p_ inside the university (2.4 pg·m^−3^ to 27.7 pg·m^−3^) were much higher than outdoor concentrations (1.1 pg·m^−3^ to 6.1 pg·m^−3^); while in Warsaw the average concentrations of Hg_p_ between indoor and outdoor environments were equal (1.4 pg·m^−3^) [29]. Greater variations in the concentrations of Hg_p_ over the measurement period were noted in Gliwice, and can be observed by comparing the values of geometric standard deviations of the averaged 24-h Hg_p_ concentrations from both sites.

### 3.2. Results from the Exposure Study

The goal of this study was to evaluate the potential cancer risk from exposure to indoor and outdoor PM_1_-bound PAHs and Hg_p_ for students from two Polish universities, taking into account the activity patterns of students calculated on the basis of statistics from a U.S. federal survey. Table 4 displays the inhalation concentrations/doses and health risks from harmful PM_1_-bound compounds. The daily inhalation exposure level (IEL) values calculated as the sum of exposure to 16 priority PAHs ranged from 26 ng·day^−1^ (Warsaw University) to 67.94 ng·day^−1^ (Gliwice University). Low I/O values for benzo(a)pyrene equivalent (BaP_eg_; ng·m^−3^) in Gliwice (0.85) and Warsaw (0.83) confirm the contribution of outdoor emissions from residential heating and coal plants in the case of Gliwice and the effects of adjacent road traffic in the case of atmospheric PAH levels in Warsaw [51].

The potential ILCR of students exposed to PM_1_-bound PAHs ranged from 5.49 × 10^−8^ (Warsaw) to 1.43 × 10^−7^ (Gliwice) (Table 4). Our results suggest that the inhalation risk for students during the 5-year period of study of students was below the acceptable risk level of 1 × 10^−6^ for all sampling sites and much lower than the priority risk level (1 × 10^−4^). Higher ILCR values were found for the Gliwice students where the highest concentrations of ∑16PAH were obtained. The same calculations were performed for teaching staff with 40 years of work experience. In that scenario, the ILCR values were slightly increased and varied between 4.39 × 10^−7^ (Warsaw) and 1.16 × 10^−6^ (Gliwice). As in the case of the students, lecturers with prolonged (40-year) exposure are expected to have low PM-bound PAH inhalation exposure risk. Table 4 also presents the non-carcinogenic risks from toxic mercury via inhalation, including site-specific variations. The lowest HQ was observed in the case of PM_1_-bound Hg_p_ in Warsaw (3.47 × 10^−5^) and highest HQ was observed among the Gliwice students (1.3 × 10^−4^).

## 4. Discussion

**Figure 1:**
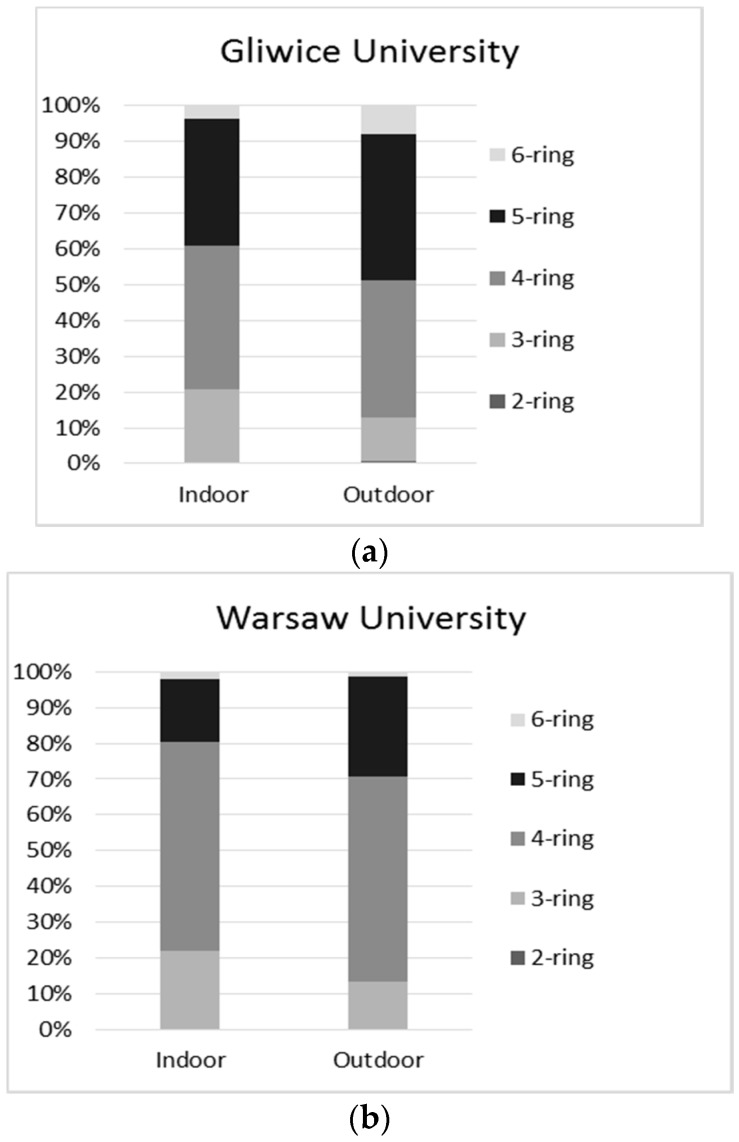
PAH distribution pattern in PM_1_ samples from lecture halls at the sites in Gliwice (**a**) and Warsaw (**b**).

It was found that summer levels of PM_1_-bound PAHs presented in this work are much lower than the winter levels presented in numerous previous works [30,50,52]. This is in accordance with the claim that the energy production for heating purposes in Poland is, in fact, the main source of fine PM. In the cities of southern Poland where the ambient PM originates mainly from coal and biomass combustion, and to a lesser extent industry, a rise in ambient concentrations of PAHs is noticeable mostly in winter [30,50,52]. In Warsaw, the capital of Poland, a large proportion of PM, even during periods when heating is extensively used, comes from traffic emissions [51]. In this work, the influence of traffic emissions on PAH levels was observed at both locations. Previously published data indicate that the combustion of fuel in gasoline and diesel engines is likely to produce high molecular weight PAHs, such as B(b *+* k)F and B(a)P [53], which may elucidate the high ambient concentration of B(b *+* k)F at the Silesia University campus (Gliwice), located next to DTŚ highway. However, the high mean concentration of PM_1_-bound FL (Table 3) and, as a result, the higher mass contribution of four-ring PAHs to ∑PAH in Warsaw (Figure 1) also reflects the road traffic effects on the PM_1_ levels [30]. When comparing the obtained results with data from the literature, it can be seen that PAH concentrations presented in Table 3 are generally much lower than the levels of outdoor PM-bound PAHs collected in other regions of the world. For example, in the south campus of Beijing Normal University in Haidian District, the sum of PM_2.5_-bound 16∑PAHs was estimated to be 150 ng·m^−3^ [54]; in the indoor air of primary schools in Lithuania, this value ranged from 20.1 ng·m^−3^ to 131 ng·m^−3^ [55]; and in primary schools in the Oporto Metropolitan Area (north of Portugal) the corresponding value ranged from 19.5 ng·m^−3^ to 82.0 ng·m^−3^ [56]. However, it is necessary to point out that study designs varied greatly in the mentioned works, namely in terms of the sampling campaign period. All of the mentioned studies were performed during the winter season, which certainly influences the significance of comparisons between the presented and discussed studies. Previously, we found that the concentrations of particulate PAHs were generally lower in the summer and spring seasons compared to those in winter at the same sites [35,50]. This suggests that the exposure values calculated in this study could be even higher if PM sampling were to be done during the heating periods.

Figure 1 presents the distribution pattern of PM_1_-bound PAHs collected in the indoor environment of lecture halls in the Gliwice (a) and Warsaw (b) universities. It is well known that the distribution of LMW and HMW PAHs depends on the physical characteristics of the compounds, as well as on the physical conditions of the studied environments, such as temperature and relative humidity. The share of HMW PAHs in their total mass found in the lecture halls of Gliwice and Warsaw universities, accounting for about 78–79% of the total PAH concentrations, was, for example, much greater than the shares found in the dust from lecture theaters in Shanghai where the values were estimated to be 30–58% [25]. This is in good agreement with previous studies indicating that low molecular weight PAHs predominantly occur in the gaseous phase, while high molecular weight PAHs tend to adhere to the particle phase [57,58]. The latter is preferable for fine particles [59,60]. This is rather obvious since HMW PAHs are characterized by low vapor pressure and very low aqueous solubility and, therefore, have a greater tendency to adhere to solid particles [61]. In total, the HMW PAHs accounted for 87% of the ∑PAHs in the outdoor environment around the universities. Any difference in the distribution of PAHs between the indoor and outdoor environment, in fact, depends on the differences in the anthropogenic sources of PAH contamination [62,63]. Overall, these results have confirmed previously reported findings from molecular diagnostic ratio (MDR) analyses that PAHs in lecture halls in Gliwice and Warsaw originate primarily from outdoor PM sources such as coal and biomass combustion or the combustion of liquid fuels in car engines [50]. The emission source of PAHs influences not only their ring number distribution but also the differences in PM size fractions. In urbanized areas of Poland, most PAHs have a size fraction in the PM_1_ category (e.g., [30,34,52] and outdoor road traffic is a strong source of PM_1_ indoors [29,30]. With regard to PM_1_, Buonanno, el al. showed that the PM_1_ number concentration is more pronounced near major freeways and in areas heated by individual heating systems [64,65]. The second source is mostly due to the emission of PAHs from coal combustion for heating purposes, which is characterized by low quality and poor efficiency [66]. Such coal is still ubiquitously and largely used in Poland. This also suggests that PM_1_ should be of prime concern with respect to PAH inhalation. Other researchers [67] measured the particle size distribution of PAHs in the urban area of Tianjin (China) and found that the mid to high molecular weight (MW 202–278) PAHs are mostly associated with fine particles (with particle diameter Dp < 2.1 µm), whereas low molecular weight (MW 128–178) PAHs are distributed between both fine and coarse particles. In addition, it has been proven that the infiltration of fine PM from outdoor sources into the indoor environment decreases exponentially with distance from the emission source. This also suggests that public facilities such as schools and universities should be located far from major emission sources such as big traffic arteries. An important observation from the PAH measurements is that the BaP concentrations inside the Gliwice and Warsaw lecture halls were 2.42 and 0.85 ng·m^−3^, respectively. Our latest study indicates that the average multi-year values of BaP in Polish areas range from 1.39 ng·m^−3^ at rural sites to as high as 4.86 ng·m^−3^ in other areas, while the yearly concentration limit is 1 ng·m^−3^. We have also proven that lifelong exposure to such high concentrations of BaP could constitute an additional risk of lung cancer [66].

There is no available data on PM_1_-bound Hg levels in the areas where this study was performed. In Poland, the ambient concentration of PM_1_-bound Hg has only been studied in Zabrze, with values ranging from 7.7 pg·m^−3^ to 186.2 pg·m^−3^ [34]. When comparing concentrations found in this study with the cited works, we conclude that the levels of Hg_p_ inside lecture halls were quite low, at about one order of magnitude lower than those popularly found in Polish urban areas. While PM-bound PAHs are primarily emitted from incomplete combustion of carbonaceous fuels, primarily coal [35], PM-bound mercury originates rather exclusively from solid fuel combustion, non-ferrous metal processing, and waste incineration [66]. Therefore, the presence and persistence of Hg_p_ in the atmosphere and its dependency on seasonal changes are completely different to PAHs. According to the earlier-presented data [29], the daily concentrations of Hg_p_ (both indoor and outdoor) were significantly correlated in Warsaw; what is more, those concentrations were also correlated with the 24-h PM_1_ concentrations inside the teaching room. Therefore, we suspect that in Warsaw, the entry of outdoor-generated Hg_p_ probably influences the indoor Hg_p_ concentrations. At the site in Gliwice, which was comprised of rather airtight rooms and used artificial ventilation, this situation was quite different. There were no significant indoor and outdoor correlations between 24-h Hg_p_ concentrations and 24-h PM_1_ concentrations. High indoor concentrations of Hg_p_ in Gliwice could be explained by the differences in the room aeration habits (rather rare in Gliwice) as compared to the Warsaw University of Life Sciences, which probably resulted in the increased accumulation of Hg_p_ inside the Gliwice lecture hall. It was also concluded that in Gliwice, some part of Hg_p_ migrating into the teaching room with the outdoor PM_1_ was probably enriched with gaseous mercury, which made the indoor Hg_p_ levels relatively high. A full and detailed discussion concerning concentrations of PM_1_, PAHs, and Hg_p_ inside and outside the lecture halls can be found in [29,30] and, therefore, will not be discussed in depth in the presented work.

### Inhalation Exposure

The mean daily intake of ∑PAH_16_ and the BaP equivalent quantity (BaP_eq_), calculated based on the exposure to individual PAH compounds and toxic equivalent factors (TEFs) for students and lecturers via the inhalation pathway, is presented in Table 4. The IEL values for the students with respect to BaP-adjusted PAHs ranged from 26 ng·day^−1^ (Warsaw University) to 67.94 ng·day^−1^ (Gliwice University) and were much lower compared to extremely high daily PAH inhalation exposure values found among children studying in schools in Delhi, India, in close proximity to roads with heavy traffic where values reached 439.43 ng·day^−1^ in the winter, 232.59 ng·day^−1^ during the monsoon, and 171.08 ng·day^−1^ during the summer season [68]. The inhalation exposure values with respect to PAHs found in this work (Table 3) are, however, comparable to those found inside lecture theatres (BaP_eq_ = 0.66 ng·m^−3^) and dining halls (BaP_eq_ = 0.73 ng·m^−3^) in Shanghai’s universities [25].

On reviewing Equation (5), one can see that the indoor inhalation exposure values of PAHs in the lecture halls (due to longer exposure time spans) are probably 5-fold higher than the corresponding values in the outdoor environment. Similar to the observed pattern, Jyethi, et al. found that the children’s daily inhalation exposure to PAHs during the day exhibited a trend with respect to school hours (indoor activity) versus commuting to school (outdoor activities) [68]. It is, therefore, easy to conclude that the greater amount of time that students spend inside the university makes the indoor exposure greater than the outdoor exposure. While measuring ∑18PAHs in indoor dust samples in Shanghai’s universities, Peng, et al. found that students in dining halls had a higher exposure to PM-bound PAHs than when in lecture theatres [25]. The total concentrations of ∑18PAHs ranged from 9.84 µg·g^−1^ to 21.44 µg·g^−1^ for dust samples from lecture theatres and from 9.63 µg·g^−1^ to 44.13 µg·g^−1^ for samples from dining halls. Higher concentrations of PAHs from cooking practices, in fact, originate from coal combustion and petroleum-related sources (ovens, stoves, gas kitchens). Since the principal source of airborne polycyclic aromatic hydrocarbons in building interiors is, by far, from the combustion of organic matter, it may be suspected that their presence in the internal environments (e.g., typical lecture halls) would be rather low. This is because the internal heat inside lecture halls in most cases is regulated by convective heaters and radiators and not by open-burning sources. For example, when comparing the concentrations of PAHs adjusted with respect to the BaP toxicity equivalent, which migrates into students’ lungs during lectures (BaP_eq_ = 2.9 ng·m^−3^ in Gliwice and 1.02 in Warsaw), with the concentration of PM-bound PAHs originating from grilling food on charcoal briquettes (BaP_eq_ = 401.6 ng·day^−1^) [69], it can be observed that PAH emissions in lecture halls are practically negligible.

In the present study, we estimated the potential ILCR values for students due to exposure to PAHs over 5 years and the exposure for lecturers over 40 years (Table 4). Our results suggest that the inhalation risk to students from the universities in Gliwice and Warsaw was below the acceptable risk level of 10^−6^ (5.49 × 10^−8^ in Warsaw and 1.43 × 10^−7^ in Gliwice). For lecturers, the ILCR value for exposure to PAHs at the Gliwice site was higher than 10^−6^ but lower than the priority risk level value (10^−4^). On comparing those values with the values for life-long BaP inhalation lung cancer risk in the general population (women and men jointly) inhabiting the Mazowieckie province (central Poland; 3.0 × 10^−3^) and the Silesia province (southern Poland; 3.3 × 10^−3^), it was found that the additional risk resulting from the rather short 5-year exposure to PAHs in teaching rooms was virtually unnoticeable [66].

In all cases tested, the non-carcinogenic inhalation risks from Hg_p_ were below the maximum acceptable level (HQ = 1) revealing no potential toxic effects to the student populations. Therefore, we conclude that mercury inhalation in both indoor and outdoor environments is not relevant to students. The non-carcinogenic risks associated with particulate mercury (Hg_p_) in the case of exposure to teaching staff were also well below 1. This demonstrates that even 40 years of exposure to Hg_p_ is not likely to pose health risks.

## 5. Conclusions

The main goals of this study were to determine and analyze the indoor air quality in lecture halls in terms of PM-bound PAHs and Hg_p_ levels. The carcinogenic risks for students in terms of individual PAHs and the combined risks associated with the sum of those substances were below the maximum acceptable level (1 × 10^−6^), revealing that the carcinogenic risk posed to university students via inhalation is acceptable. In addition, the non-carcinogenic risks to students aged 21 as a result of exposure to PM_1_-bound mercury are also far below the acceptable level of HQ = 1, indicating no toxic effects. The indoor concentrations of PAHs and Hg_p_ varied between the considered university halls. Outdoor concentrations of ∑16PAHs were probably shaped by nearby traffic-related emission sources, while the Hg_p_ levels (especially in the Gliwice site) were regulated by the existence of some unidentified indoor sources enriching PM_1_ levels. It was found that the lecture hall in Gliwice, due to its airtightness and inappropriate air ventilation, was more detrimental to student health. The existing concentration differences between indoor and outdoor environments do not, however, allow us to state how strongly the indoor concentrations depend on the penetration of outdoor contaminated air. The effectiveness of staying indoors to reduce student exposure to outdoor-source PM compounds is, therefore, unknown. The results presented in this study indicate that there is no need to reduce the risks of adverse health effects from indoor air pollution inside the lecture halls. It must be noted, however, that providing appropriate indoor air conditions is an important task for educational institutions in order to ensure high productivity of its students. It is vitally important to keep indoor air conditions within permissible and healthy limits as established and determined by special indoor air quality standards.

## Figures and Tables

**Table 1:** Students’ activity characteristics [33].

Activity No	Type of Activity	Average Duration over a Day (h)	Proportion of a Day (%)	I/O Environment
1	traveling	1.4	5.83	O
2	other	2.2	9.16	O
3	grooming	0.8	3.33	I
4	eating and drinking	1.0	4.14	I
5	educational activities	3.5	14.58	I
6	working and related activities	2.3	9.58	I
7	leisure and sports	4.0	16.66	I
8	sleeping	8.8	36.66	I

**Table 2:** A list of polycyclic aromatic hydrocarbons (PAHs) with carcinogenic potency and their toxicity data.

Common Name	Abbreviation	Rings	CAS-No	Genotoxicity *	IARC Classification **	Toxic Equivalency Factor TEF ***
naphthalene	NAP	2	91-20-3	Negative	2B	0.001
acenaphthylene	AcPy	3	208-96-8	Questionable	Not evaluated	0.001
acenaphthene	Acp	3	83-32-9	Questionable	3	0.001
fluorene	Flu	3	86-73-7	Negative	3	0.001
phenanthrene	PA	3	5801-8	Questionable	3	0.001
anthracene	Ant	3	120-12-7	Negative	3	0.01
fluoranthene	FL	4	206-44-0	Positive	3	0.001
pyrene	Pyr	4	129-00-0	Questionable	3	0.001
benz[a]anthracene	BaA	4	56-55-3	Positive	2B	0.1
chrysene	CHR	4	219-01-9	Positive	2B	0.01
benzo[b]fluoranthene	BbF	5	205-99-2	Positive	2B	0.1
benzo[k]fluoranthene	BkF	5	207-08-9	Positive	2B	0.1
benzo[a]pyrene	BaP	5	50-32-8	Positive	1	1
indeno[1,2,3-cd]pyrene	IND	6	193-39-5	Positive	2B	0.1
dibenz[a,h]anthracene	DBA	5	53-70-3	Positive	2A	1
benzo[ghi]perylene	BghiP	6	191-24-2	Positive	3	0.01

* WHO (World Health Organization) Classification, 1998. International Program on Chemical Safety. Environmental Health Criteria 202, Selected Non-Heterocyclic and Polycyclic Aromatic Hydrocarbons [44]; ** IARC (International Agency for Research on Cancer) Classification Group 1: The agent is carcinogenic to humans. Group 2A: The agent is probably carcinogenic to humans. Group 2B: The agent is possibly carcinogenic to humans. Group 3: The agent is not classifiable as to its carcinogenicity to humans [45]; *** Toxic equivalency factor (TEF) for selected polycyclic aromatic hydrocarbons (PAHs) based on the toxicity of benzo[a]pyrene. In [43].

**Table 3:** The geometric mean and geometric standard deviations from the 24-h concentrations of 16 PAHs (ng·m^−3^) and Hg_p_ (pg·m^−3^) in the indoor (I) and outdoor (O) environments of a specific university.

Number	Symbol	Rings	Gliwice University				Warsaw University			
			PAHs (ng·m^−3^)		Hg_p_ (pg·m^−3^)		PAHs (ng·m^−3^)		Hg_p_ (pg·m^−3^)	
			I	O	I	O	I	O	I	O
			Geom.mean ± Geom.std	Geom.mean ± Geom.std	Geom.mean ± Geom.std	Geom.mean ± Geom.std	Geom.mean ± Geom.std	Geom.mean ± Geom.std	Geom.mean ± Geom.std	Geom.mean ± Geom.std
1	NAP	2	0.02 ± 0.02	0.12 ± 0.31	6.38 ± 6.03	3.03 ± 1.57	0.01	-	1.46 ± 0.73	1.38 ± 0.52
2	AcPy	3	0.24 ± 1.81	0.28 ± 6.94			0.14 ± 4.32	0.32 ± 2.78		
3	Acp	3	0.04 ± 0.36	0.04 ± 1.20			0.05 ± 0.29	0.11 ± 1.84		
4	Flu	3	1.11 ± 5.94	1.05 ± 1.54			0.55 ± 0.73	0.25 ± 0.30		
5	PA	3	0.18 ± 2.77	0.19 ± 3.15			0.48 ± 0.39	0.12 ± 0.26		
6	Ant	3	1.10 ± 4.05	0.23 ± 1.29			0.27 ± 0.35	0.27 ± 1.17		
7	FL	4	1.61 ± 6.70	1.56 ± 15.13			2.31 ± 6.84	2.70 ± 2.38		
8	Pyr	4	0.40 ± 0.43	0.57 ± 1.56			0.38 ± 0.34	0.17 ± 0.49		
9	BaA	4	1.60 ± 1.89	1.99 ± 2.80			0.46 ± 0.66	0.57 ± 0.60		
10	CHR	4	1.59 ± 2.65	1.57 ± 2.50			0.79 ± 2.51	1.21 ± 1.66		
11	BbF	5	1.17 ± 1.47	1.90 ± 1.24			0.21 ± 2.86	0.36 ± 0.20		
12	BkF	5	0.93 ± 1.31	1.40 ± 1.06			0.06 ± 0.71	0.26 ± 0.12		
13	BaP	5	2.42 ± 3.58	2.72 ± 2.24			0.85 ± 0.96	1.54 ± 1.01		
14	IND	6	0.15 ± 0.39	0.67 ± 10.72			0.12 ± 2.06	0.09 ± 0.79		
15	DBA	5	0.07 ± 4.78	0.04 ± 0.47			0.08 ± 6.89	0.08 ± 1.78		
16	BghiP	6	0.34 ± 3.86	0.52 ± 0.64			0.02 ± 0.15	0.03 ± 0.25		
∑16PAHs (ng·m^−3^)			12.97	14.85			6.78	8.08		
BaP_eq_ (ng·m^−3^)			2.90	3.38			1.02	1.76		

PAH: polycyclic aromatic hydrocarbons; NAP: naphthalene; AcPy: acenaphthylene; Acp: acenaphthene; Flu: fluorene; PA: phenantrene; Ant: anthracene; Fl: fluoranthene; Pyr: pyrene; BaA: benzo[a]anthracene; CHR: chrysene; BbF: benzo[b]fluoranthene; BkF: benzo[k]fluoranthene; BaP: benzo[a]pyrene; IND: indeno[1,2,3-cd]pyrene; DBA: dibenzo[ah]anthracene; BghiP: benzo[ghi]perylene; Hg_p_: particulate bound mercury.

**Table 4:** The inhalation doses of PM_1_-bound Hg_p_ and PAHs and the related non-carcinogenic and carcinogenic health risks at Polish universities.

	Gliwice University	Warsaw University
IEL_students_ ng·day^−1^	67.94	26.0
EC_students and lecturers_ µg·m^−3^	1.41 × 10^−4^	3.47 × 10^−5^
ILCR_students_	1.43 × 10^−7^	5.49 × 10^−8^
ILCR_lecturers_	1.15 × 10^−6^	4.39 × 10^−7^
HQ_students and lecturers_	1.3 × 10^−4^	3.47 × 10^−5^
